# Exploring Stiffness Modulation in Prosthetic Hands and Its Perceived Function in Manipulation and Social Interaction

**DOI:** 10.3389/fnbot.2020.00033

**Published:** 2020-06-25

**Authors:** Patricia Capsi-Morales, Cristina Piazza, Manuel G. Catalano, Antonio Bicchi, Giorgio Grioli

**Affiliations:** ^1^Centro “E. Piaggio” and Dipartimento di Ingegneria dell'Informazione, University of Pisa, Pisa, Italy; ^2^Istituto Italiano di Tecnologia, Genova, Italy

**Keywords:** prosthetics, impedance control, soft robotics, human-robot social interaction, task adaptability

## Abstract

To physically interact with a rich variety of environments and to match situation-dependent requirements, humans adapt both the force and stiffness of their limbs. Reflecting this behavior in prostheses may promote a more natural and intuitive control and, consequently, improve prostheses acceptance in everyday life. This pilot study proposes a method to control a prosthetic robot hand and its impedance, and explores the utility of variable stiffness when performing activities of daily living and physical social interactions. The proposed method is capable of a simultaneous and proportional decoding of position and stiffness intentions from two surface electro-myographic sensors placed over a pair of antagonistic muscles. The feasibility of our approach is validated and compared to existing control modalities in a preliminary study involving one prosthesis user. The algorithm is implemented in a soft under-actuated prosthetic hand (SoftHand Pro). Then, we evaluate the usability of the proposed approach while executing a variety of tasks. Among these tasks, the user interacts with other 12 able-bodied subjects, whose experiences were also assessed. Several statistically significant aspects from the System Usability Scale indicate user's preference of variable stiffness control over low or high constant stiffness due to its reactivity and adaptability. Feedback reported by able-bodied subjects reveal a general tendency to favor soft interaction, i.e., low stiffness, which is perceived more human-like and comfortable. These combined results suggest the use of variable stiffness as a viable compromise between firm control and safe interaction which is worth investigating further.

## 1. Introduction

**Figure 1:**
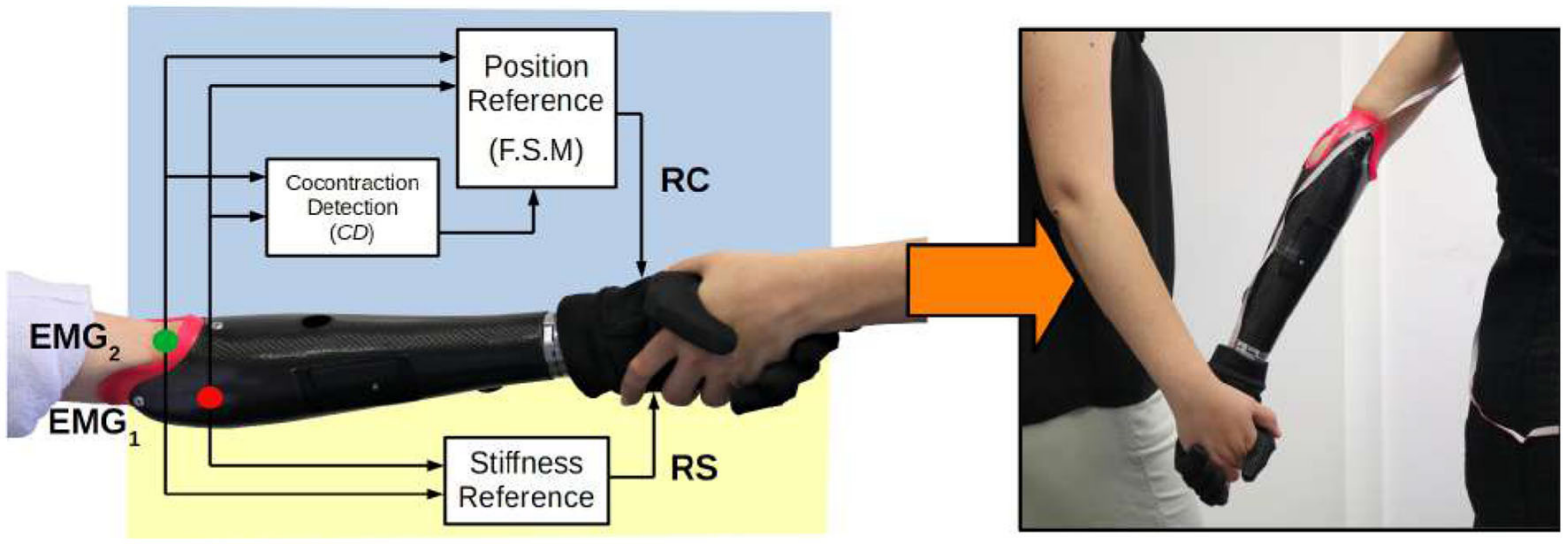
Block diagram of the proposed method. Three functional blocks decode stiffness and position references from a pair of antagonistic sEMG sensors. The introduction of variable stiffness control in a soft robotic hand allows a more natural physical social interaction between two subjects.

An upper limb amputation leaves a person with limited ability to perform work and daily living activities, but also hinders social interaction and the perception of self-image (Atkins et al., 1996). Artificial limbs are a valuable tool to restore some of these lost capabilities. However, there is still a sharp separation between what functional devices available today can offer and what prosthesis users really need. The quality and safety of Human-Robot interaction, are aspects that cannot be underestimated in prosthetics, especially in upper limb, due to the inherently interactive nature of the (artificial) hand. Impedance control, which plays a pivotal role in human movement, could be tantamount to the promotion of natural bionic interaction (see Figure 1).

In the context of robotic manipulation, impedance control was introduced about 35 years ago by Hogan (1985). It led to a revolution in how modern robots are controlled (De Luca et al., 2006), and in what they are able to accomplish (Hochberg et al., 2012), paving the way to collaborative robotics (Haddadin et al., 2017). Today Social Human-Robot Interaction is an emerging field of investigation. It gave rise to substantial research, including studies on grip force control of robotic hands interacting with humans (Garate et al., 2018; Vigni et al., 2019). Furthermore, examples like Duffy (2003) and Cramer et al. (2009) suggest that autonomy, adaptability and touch are key requirements for social robots to be seen as less machine-like by humans. Human impedance-regulation skills have been transferred to robots (including hands) in the framework of teleimpedance (Ajoudani et al., 2013).

Already in Hogan (1983), the author suggested impedance control as the preferred paradigm for controlling prostheses. It would provide the amputee with an essential component of the natural adaptative capability of humans, which would be difficult to recover otherwise, due to inherent severe sensory loss. This approach was investigated by Sensinger et al. (2008), whose experiments led to the conclusion that proportional velocity control of the joint position and high values of impedance obtain faster and more precise task execution. Their results also suggested a possible inability to fine-tune the impedance when using EMG signals, because of their noisy nature. However, some years later, impedance controllers proved to feature a more natural control, to be easier to use, and to enhance user's experience (Tsuji et al., 2000). Moreover, Blank et al. (2014) proved the existence of task-dependent optimal values of stiffness, a result in agreement with the literature about muscles stiffness control (e.g., Flanagan and Wing, 1997), which proves that different tasks require different properties. Interestingly, subjects in Blank et al. (2014) were able to recognize the difficulties in performing the tasks, in accordance with the stiffness value implemented on the system.

**Figure 2:**
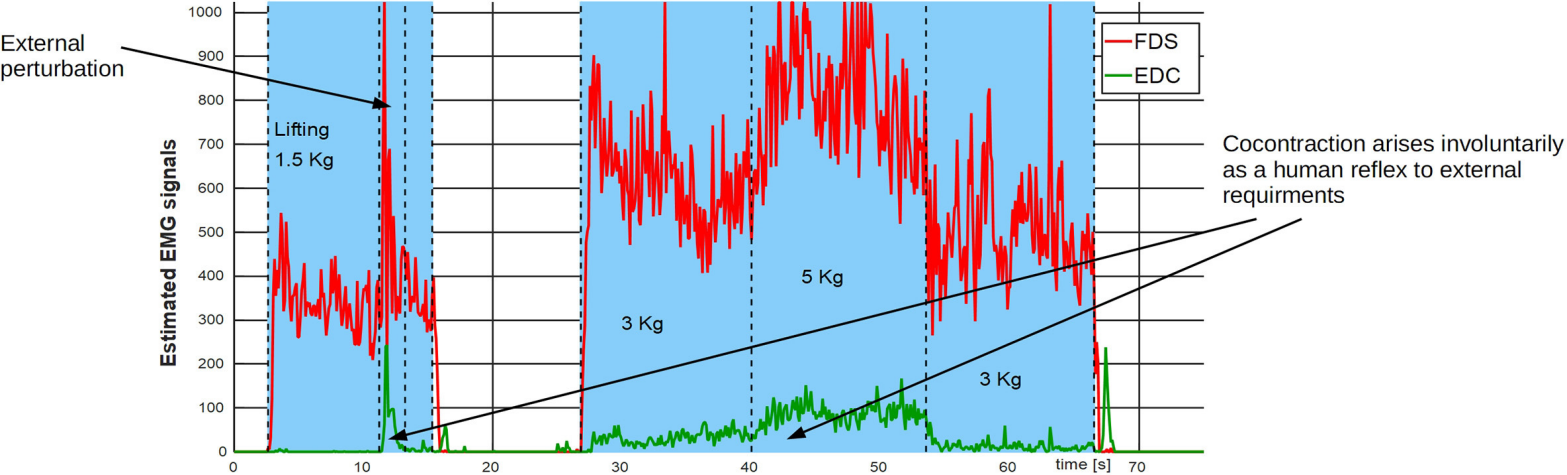
Natural behavior of a pair of antagonistic muscles subject to different load conditions. Data is extracted from one major antagonistic group of muscles acting on the wrist (FDS and EDC) of an able-bodied subject (female, age 32). Muscle coactivation is present when the subject is lifting heavier objects (3–5 kg) and as a reaction to an external perturbation when lifting a lighter load (1.5 kg).

Unfortunately, control research advancements struggle to be translated into prosthetic applications, due to motivations that include, among others, the lack of intuitiveness and robustness (Farina et al., 2014). This issue highlights the importance of designing control strategies that enhance the user's experience, a goal that, in our particular case, is strictly connected to finding a natural description of muscle stiffness modulation. Behavioral studies of postural limb control show that humans modulate joint stiffness to minimize the perturbing effects of external loads (Latash, 1992), or to minimize interaction torques (Gribble and Ostry, 1999). This was proven to benefit limb stability and movement accuracy (Gribble et al., 2003). It is well-known that the position of a joint is defined by the equilibrium of the various muscles acting on it, together with external forces. However, the concurrent action of antagonistic muscles, i.e., coactivation, defines the mechanical properties of the joint as well. There is evidence (Gribble and Ostry, 1998) that coactivation increases with movement velocity and with the magnitude of perturbing forces, and that it decreases gradually over the course of learning a novel motor task (Osu et al., 2002). Although metabolically expensive, in the presence of noise, the compromise between energy consumption and postural positioning error does favor unintentional antagonist muscle coactivation (Hogan, 1984). Figure 2 presents an example of this natural tendency in humans when lifting heavy objects or reacting to external perturbations.

Myoelectric control of prostheses has been traditionally implemented following the paradigm of direct control, a rather old technique that affirmed over the years (see Fougner et al., 2012 for a review), to the point of becoming a standard in the market. This technique uses the intensity of the EMG signals, extracted from a pair of antagonistic muscles, to generate reference commands to operate a myoelectric hand along two opposing motion directions: one EMG associated to closing, and the other associated to opening (see section 2 for a detailed discussion). Within the framework of standard direct control, muscle cocontraction is, in first approximation, considered as a source of noise to be discarded. Nevertheless, over the years, control engineers devised algorithms to enable prosthesis users to voluntarily use cocontraction as an additional control input. Nowadays, there is the possibility of using isolated cocontraction events to switch the operation of poly-articulated commercial hands between different motion patterns, along which the prosthesis is then operated via direct control (Roche et al., 2014). Most recently, several examples in literature uses motion classes for poly-articulated hands, based on the recognition of muscle coactivation patterns (e.g., Samuel et al., 2018; Asogbon et al., 2020).

However, in natural conditions, the human neuromotor system performs a more active and rich use of muscle cocontraction. Indeed, muscle activation controls, proportionally and simultaneously, joint torques and speeds, limb positions and impedance, and balances contact forces (Hogan, 1983; Fougner et al., 2012). This encouraged the design of control methods that use multiple EMG signals from groups of muscles to operate, simultaneously and proportionally, more than one degree of freedom. However, the number of independent EMG signals that is possible to extract from the muscles of an amputee is generally very small. This is due to both the clinical condition of the muscles and the technical difficulty of fitting many sensors in a small space. Novel techniques have been proposed to overcome this issue (e.g., Sartori et al., 2013; Maimeri et al., 2019), where muscle information is integrated within a musculoskeletal model or with postural measures.

Inspired by the natural behavior of muscles, we consider the possibility of implementing a simultaneous and proportional control method to command both the position and the stiffness in prosthetics. We believe that the implementation of these capabilities would improve control naturalness of prosthetic hands, promote bionic interaction, and in turn, favor their acceptance. Actually, artificial hands are already integrated into everyday life of prosthesis users, but little is known about their capability to interact socially and to adapt to situation-dependent requirements. In this paper, we aim at investigating the role of variable stiffness control in prosthetic hands for completing ADL and social interaction.

We present a method capable of decoding both stiffness and position intentions from one pair of antagonistic muscles (using two sEMG sensors). Even though our work is inspired by Sensinger et al. (2008), there are some important differences on the proposed method (discussed in detail in section 3). Our algorithm aims at describing user's stiffness modulation, proportional to muscle coactivation, and closure/aperture speed, proportional to reciprocal activation through the use of a Finite State Machine.

Although the use of cocontraction to control impedance could compromise the standard adoption of this feature as a switching technique, it is important to stress the existence of other available options in the market for switching, e.g., sequences of time-based contractions, external inputs, etc. Moreover, it is true that not all prosthetic systems need switching to operate. One example of this is the common tridigital myoelectric hand, while another is the soft-underactuated prosthetic hand (SoftHand Pro–SHP–Godfrey et al., 2018) that we used as our experimental platform. The SoftHand Pro, whose design is based on the concept of soft synergies (Catalano et al., 2014), combines the implementation of a single motor function, inspired by the first synergy of human grasping (Santello et al., 1998), with the intrinsic softness of its 19 degrees of freedom, to adapt its grasp pattern to the case-dependent contact constraints.

The proposed method was implemented on the SHP and tested by a prosthesis user (see Figure 1). To the best knowledge of the authors, this is the first experimental validation of the feasibility of mechanical impedance control in prosthetic hands, performed by an amputee. In fact, both Sensinger et al. (2008) and Blank et al. (2014) tested their proposed approaches with able-bodied subjects only. We asked the user to rate the control system usability after performing a set of tasks, which include one- and two-handed object manipulation, self-interaction, and social interaction with 12 able-bodied volunteers. Volunteers feedback was also collected and analyzed. Encouraging results evidence that variable stiffness could be a possible compromise between modalities that just favor either firm control or delicate interaction. This insight suggests to extend this preliminary study by including multiple subjects with limb loss and different robotic hands.

This paper is organized as follows: section 1 introduces the concept and motivation for using impedance control in prosthetics, while section 2 presents its background. We discuss the implementation of the proposed algorithm in section 3 and its validation in section 4. Section 5 describes the prosthesis used in two experimental studies. First, in section 6 we study the feasibility of the algorithm with a prosthesis user and compare its performance with standard control methods. Then, in section 7, we evaluate the use of variable stiffness and grasp perception from one prosthesis user and 12 able-bodied subjects interacting with it. Finally, section 8 shows the main outcomes of the article and summarize our contribution.

## 2. Background

Among functional hand prostheses, the role of myoelectric hands is particularly important (Roche et al., 2014). Users operate the device directly with their muscle signals, which favors the perception of the robotic aid as an extension of their body. Classically, the problem of controlling commercial myoelectric hands reduces to the issue of determining a reference configuration value (*RC*) from the online manipulation of a pair of sEMG signals, recorded from two antagonistic muscles. Nonetheless, several theories in motor control try to explain the neurophysiological variables and their dynamic relation to define both position and stiffness commands in natural conditions. Feldman (1986) introduced the idea that considering a single muscle, setting the threshold value of the tonic stretch reflex (λ) leads to a dependence of active muscle force on muscle length (an invariant characteristic function, IC). The definition of λ emerges from external load characteristics. The intersection point between the load and IC is the equilibrium point (EP) of the system (Feldman and Latash, 2005). Either passively or actively varying λ, which is linked-to the stiffness, and changes with antagonistic coactivation, the original EP shifts to a new location that may involve a change in length, force, or both. Notwithstanding the importance of external loads in object manipulation, it is fundamental to notice that this factor is not always considered in the control of hand prostheses.

### 2.1. Decoding Position Intentions

In conventional systems, the simplest way to control one Degree of Freedom (DoF) prosthesis is by the so-called proportional position control approach (PPC), where the motor voltage is proportional to the difference between the EMG signals amplitude (Sears and Shaperman, 1991). In modern prostheses, electrodes do usually include preamplification and analog-to-digital conversion. Sometimes they also embed signal conditioning (e.g., rectification and low-pass filtering) to prepare the signal for feature extraction (Fougner et al., 2012). EMG signals are usually further processed with an activation threshold to remove extra noise. PPC is considered very flexible and user-friendly in subjects with transradial amputations. However, PPC requires continuous muscle activation to hold the prosthesis in any position different from that associated with null EMG signals. This is an important limitation in prosthetics, because continuous muscle activation tends to tire users (Biddiss and Chau, 2007). To overcome this limitation, it is very common in the practice to use the same command to drive the speed of *RC*, rather than *RC* itself. Proportional velocity controls (PVC) define *RC* proportional to the integral of the EMG signals difference. Here, users can relax their muscles when the hand is closed and just activate them to move the hand in aa explicit direction (closing or opening). An additional feature is the reduction of involuntary activations due to external perturbations, to which PPC is more sensitive.

Nonetheless, also PVC presents some drawbacks in presence of coactivation, e.g., during very quick movements (Gordon and Ghez, 1984). In fact, since it is natural to observe some level of cocontraction in EMG patterns of fast motions, a purely integral control can have the inconvenience of incorrectly mapping intentions of fast motions into slower ones. Indeed, when both EMG signals activate at the same time, their difference can result in a low or null value, canceling the activation of the system, contrarily to user's intended action. For this reason, a Finite State Machine (FSM) can be used to select which EMG signal should command the system according to the detection of a motion class, rather than using signals difference.

### 2.2. Decoding Stiffness Intentions

While standard high-level control systems use EMG signals only to reconstruct a position reference, more sophisticated low-level control paradigms, as mechanical impedance control (Hogan, 1984) can be used to determine the dynamic relation between manipulator variables, such as end-point position and force, to adapt the system performance to different requirements. It is known that even a simple implementation of this approach, such as the active tuning of the proportional gain (*Kp*) of the motor controller, can strongly modify the performance of a robotic system (e.g., Fu and Santello, 2018). This tuning affects both the action velocity, e.g., closing or opening, and the grip rigidity (i.e., hand stiffness).

In the recent past, Sensinger et al. (2008) analyzed the effect of impedance control on a single degree of freedom prosthetic elbow controlled by able-bodied subjects to execute fine positioning tasks. They investigated the appropriate baseline impedance values of the system, and the strategy that obtains the best performance for joint position control. After this, they tested the voluntary modulation of different impedance parameters while performing the task, and their relation with accuracy.

Teleimpedence Based Control Strategy (Ajoudani et al., 2013), mostly used in robotics, incorporates both postural and stiffness intentions in real-time through proportional control systems; where *RC* is determined with EMG signals difference (in a PPC-like fashion), and the stiffness with their sum. When adjusting task-related grasp forces with an impedance controller, lower cocontraction results in higher compliance, allowing gentle grasping of fragile or deformable objects. Instead, higher stiffness values are experienced when grasping heavier or more rigid objects. This feature enables smooth modulation of the hand estimated force, while still allowing for task completion (Godfrey et al., 2013). Similar approaches are often used for the control of exoskeleton joints (e.g., Lenzi et al., 2011; Proietti et al., 2016).

Finally, Blank et al. (2014) studied if user-selectable impedance prostheses could improve the user's ability to interact effectively with a variety of environments. They present two different type of tasks: (1) a force minimization task, which is related with soft interaction, and (2) a trajectory tracking task, associated with fine positioning or accuracy. They investigated how the variation of different stiffness and damping levels affects on able-bodied subjects performance in a virtual environment, and then, on the control of a robotic arm. This work identified areas in which non-ideal physical characteristics of the prosthetic system could limit users' performance.

## 3. Simultaneous and Proportional Extraction of Position and Stiffness Commands

Although several investigations confirm the possible advantages of impedance control in prosthetics, its practical application is hindered by the choice of the particular strategy adopted for the generation of both stiffness and postural references. Motivated by the state of the art presented above, we want to investigate the use of impedance control to enhance the prosthesis functionality in the execution of different tasks. Moreover, we want to promote a control strategy that, while trying to be as natural and intuitive as possible, does not increase the risk of fatigue or mental effort (Østlie et al., 2012). Therefore, we propose a method, based on the combination of three functional blocks, to define two simultaneous and proportional references for position and stiffness (see block diagram in Figure 1).

It is important to clarify that in this paper we do not mean to measure nor reconstruct position and stiffness from EMG values. We propose the definition of two indices inspired by the relationship between EMG signals and limb position and stiffness in humans. Then, we use these indices to instrumentally generate reference commands for a variable impedance prosthesis.

Moreover, notice that, although our approach aims at reducing the risk of fatigue, it does not measure nor control fatigue explicitly. This is a drawback shared with most of direct control techniques, due to the difficulties in distinguishing fatigue from low muscle activation with commercial sEMG instrumentations. Moreover, since the functional relationship between the user's inputs and the control system outputs must be of a suitable form (Fougner et al., 2012), the parameters of the proposed myoelectrical algorithm should be personalized for each patient condition, to be capable of rejecting low interferences while being sensitive to user's intended actions. While this paper is limited to a pilot feasibility study of the proposed approach with one prosthesis user, further investigations on fatigue effects and personalization are deferred to future work.

### 3.1. Stiffness Command

Inspired by the research of Ajoudani et al. (2013) and Blank et al. (2014), we control the stiffness of a prosthetic hand though the active tuning of the proportional gain of the motor controller. This provides the system with low stiffness, which could be useful for collaborative tasks and soft interaction, or high stiffness, which could reject perturbation and obtain safer grasps (e.g., in the case of heavy objects) depending on the user.

We construct the stiffness command based on the definition of the Stiffness Index (*Is*). *Is* is proportional to the total activation of user's muscles and it is calculated as the weighted average of the level of activation from two antagonistic muscles (as in Ajoudani et al., 2013). Therefore, *Is* and the stiffness command can either increase due to an involuntary reaction to external disturbances, voluntary cocontraction, or due to muscle contraction. This enables the system not to avoid losing reactivity when moving the reference configuration, and to perform different grip behaviors. The use of this definition promotes the reproduction of natural coactivation, which also replicates user's reflexes, i.e., involuntary reactions against external perturbations. This feature does not increase the user's mental effort, as the system naturally adapts according to the state of user's muscles without explicit voluntary activation. Moreover, defining *Is* as the average antagonistic activation ensures the existence of some amount of impedance modulation also when cocontraction is limited, as it could occur in amputees.

### 3.2. Position Command

Inspired by Sensinger et al. (2008), who shows superiority of velocity control over position control, also when modulating impedance, we pursue proportional velocity control (PVC) for hand position commands. Indeed, this strategy is also the preferred one in commercial devices to reduce the risk of fatiguing users. With respect to Sensinger et al. (2008), we include a Finite State Machine (FSM) to properly avoid interference of involuntary and voluntary cocontractions, which are associated with user's stiffness modulation, with positions commands.

**Figure 3:**
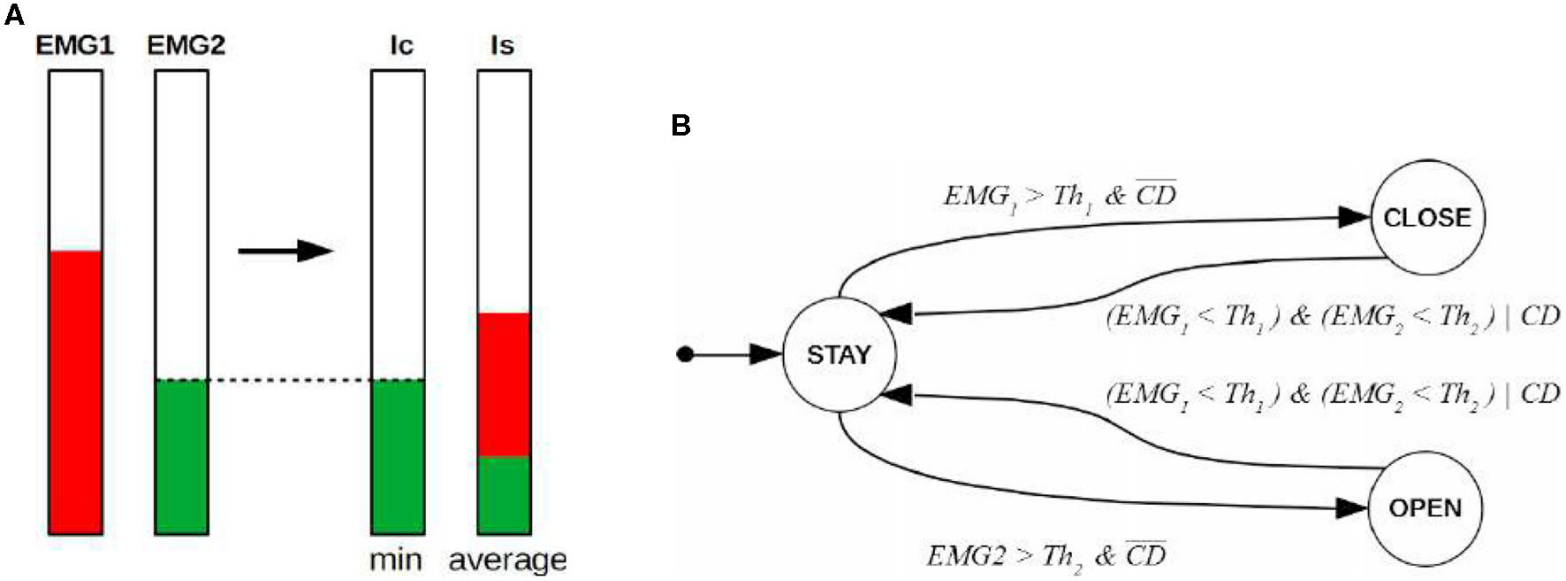
Proposed method. **(A)** Shows a schematic representation of the variables involved in coactivation. The quantity *Ic* defines *CD* as explain in Equation (2). **(B)** Presents the Finite State Machine used to refine the speed of the hand. Hand states are defined by circles, while guard conditions are written directly nearby the arrow connecting pre and post states. The starting state is *STAY*. *Th*_1_ and *Th*_2_ are the activation threshold for each channel to detect intention of movement and reject low interferences. Note that when *CD* is false, the guard conditions of this FSM correspond to those of standard FSM used for PVC.

To properly decouple user's stiffness intentions from position commands, we introduce and monitor the cocontraction index (*Ic*) to isolate voluntary cocontraction in a binary (true/false) detection signal *CD*. In particular, *Ic* is defined as

(1) $$\begin{array}{l} Ic=\min\left(C_{1}EMG_{1},C_{2}EMG_{2}\right), \end{array}$$

where *C*_*i*_ is a suitable normalization weight for each signal. We observe that *Ic* is related to coactivation similarly to *Is*, but compared to *Is*, *Ic* is more sensitive to the lower activation levels (Feldman and Latash, 2005). Combining this with the fact that, due to the PVC approach, the level of the extensor muscle contraction is almost zero when the user is commanding closure (and the converse when opening), we detect cocontraction by defining

(2) $$CD=\{\begin{array}{ll} 0 & \text{if }Ic<ThCD\\ 1 & \text{otherwise}, \end{array}$$

where *ThCD* is a suitable threshold value. With suitable tuning of parameters, it is possible to use *CD* for the detection of cocontraction (Rao et al., 2010), as both sEMG channels have a high level of activation. Figure 3A presents a visual representation of *Ic* and *Is* indices.

The FSM used to discriminate the user's intended actions is introduced in Figure 3B. It has three states corresponding to three different possible motions of the hand: Staying still (STAY), closing (CLOSE), and opening (OPEN). Its state transitions consider the two sEMG values and *CD* as inputs. Without the latter input (i.e., *CD*), pure cocontraction phenomena, unless perfectly symmetrical and synchronized (which never happens in practice), would be interpreted as either open or close command, depending on which of the two EMG signals is observed overcoming its activation threshold first.

The state transitions regulate FSM operation and, when met, let the system jump from the present state to another. The definition of the hand *RC*, in analogy with typical velocity control frameworks, is updated according to the differential equation,

(3) $$\dot{R}C=\{\begin{array}{ll} C_{1}EMG_{1} & \text{if }CLOSE\\ 0 & \text{if }STAY\\ C_{2}EMG_{2} & \text{if }OPEN. \end{array}$$

Note that the calculation of *RC* and *RS* are simultaneous and independent, thus the algorithm keeps generating commands of position and stiffness simultaneously. It is possible to observe that there is some correlation between motion commands and stiffness, since when the user contracts one muscle, e.g., to close the hand, the stiffness command will always be larger than the minimum it has at rest, imitating the natural behavior of muscles, which is a desired and welcome effect.

## 4. Algorithm Validation

In this section, we present an example of the behavior of the proposed algorithm, and compare it to existing methods, highlighting the different interpretation of the user's intended actions. In this case, the EMG data were collected from a healthy subject (female, age 26). Two commercial sEMG sensors (13E200 = 60, OttobockGmbH, Germany) were used to acquire the inputs from the subject. The sensors, commonly used in commercial prostheses, include base signal conditioning to reject electric line noise, amplify, and rectify the signal. The onboard sensing was achieved using a custom electronic board (see Della Santina et al., 2017 for details), which is connected to a lab computer running MATLAB Simulink (Mathworks, Inc). The same signal acquisition setup was used in all the experiments described also later in the paper.

Suitable calibration of parameters is done before signal recording, to set the maximum and minimum levels of contraction of the subject, and to tune the values of the different activation thresholds and gains.

**Figure 4:**
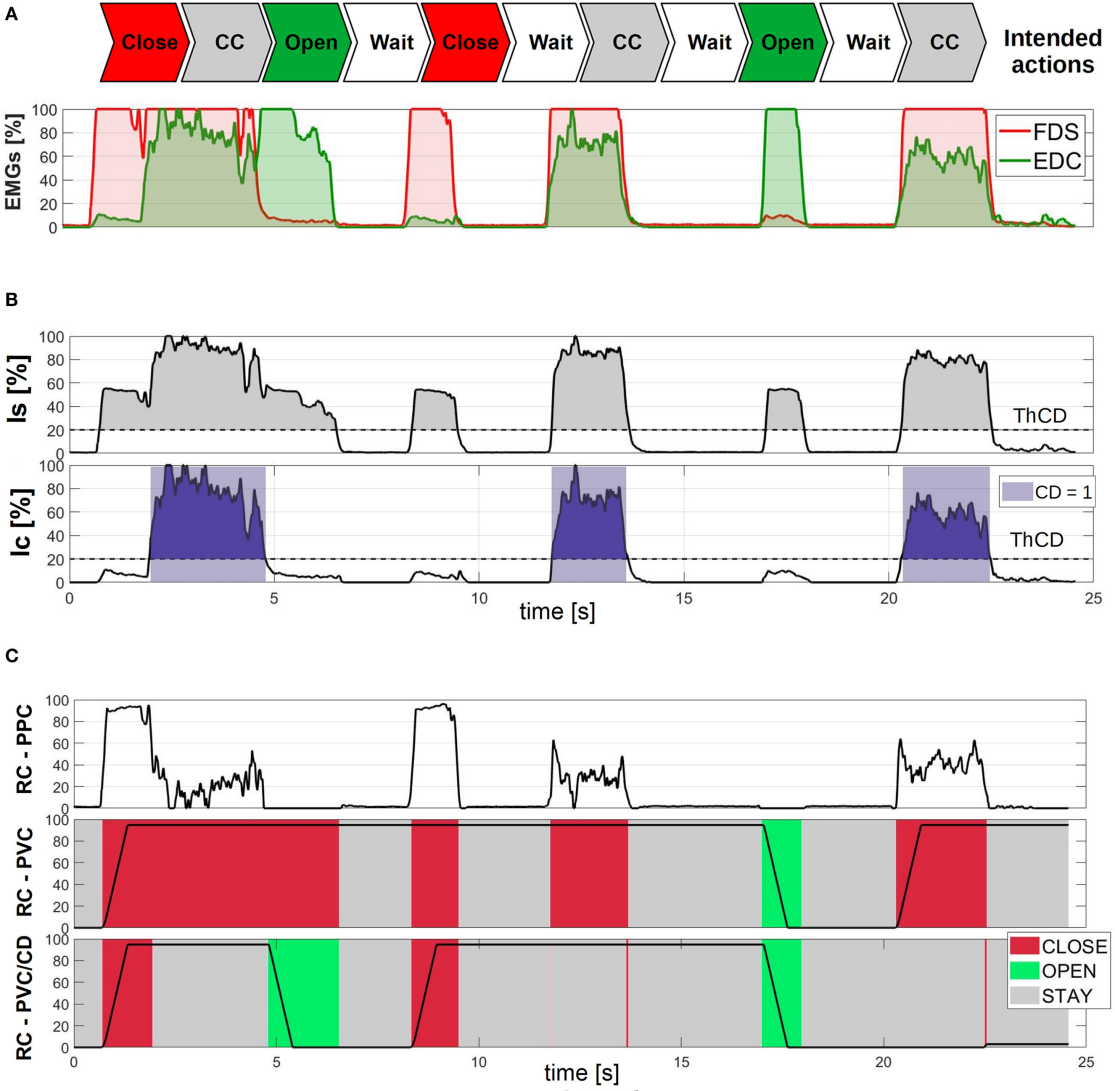
Experimental description of the variables involved in the algorithm. **(A)** Presents the estimated EMG signals from a healthy subject performing a series of intended actions. **(B)** Explains the indices related with cocontraction. There, the colored areas corresponds to the instants detected when comparing the index to a threshold *ThCD*. In **(C)**, we show an experimental comparison of the reference configuration of the hand described by different control strategies with high constant stiffness. In the two bottom cases, PVC is implemented with two different FSMs (RC-PVC/CD corresponds to the proposed method, where CD is an additional input in the state transitions). Colors represent the state (user's detected intended action) in each moment. Red areas corresponds to when closing is detected by the algorithm, green when opening, and gray when the hand keeps the previous position. All quantities are normalized to the maximum value of each variable and expressed in percentage.

Figure 4A presents the intended actions performed by the subject (top) and the corresponding EMG activation of FDS and EDC (bottom). Note that the sequence of intended actions was decided in advance, and then executed by the subject without any explicit timing.

Figure 4B shows the reconstruction of the two indices *Is* (top) and *Ic* (bottom) as extracted from the signals of Figure 4A. It is possible to notice how the stiffness index *Is* tends to rise both in the case of cocontraction and of pure contraction. This behavior reflects the natural behavior of antagonistic muscles, but complicates the detection of pure cocontraction phenomena, if it was based on the value of *Is*. This intuition motivates the introduction of the index *Ic* (bottom panel), which correlates with pure cocontractions more accurately. For this reason, we compare it with a threshold *ThCD* to reconstruct the binary cocontraction detector *CD*.

Figure 4C shows a comparison of three different approaches for the generation of the reference command *RC*, applied to the same signals of Figure 4A. The three methods are:

- **PPC:** Proportional Position Control (top);
- **PVC:** Proportional Velocity Control with the use of FSM without explicit coconctraction detection;
- **PVC/CD:** Proportional Velocity Control with explicit coconctraction detection *CD*, as extracted from the bottom panel of Figure 4B.

Note that method PVC uses a finite state machine with the same states and transition of case PVC/CD, but which does not considers *CD*. This is the same as using the finite state machine shown in Figure 3B and considering *CD* = 0.

PPC (Figure 4C top), being based on the difference between *EMG*_1_ = *FDS* and *EMG*_2_ = *EDC*, has the problem of interpreting cocontraction as if it was a reduction in the level of activation, a behavior which can be in opposition to what is intended by the user. This could result in an opening of the hand when a stiffening or a faster motion in the opposed direction is being intended. In addition, although PPC is more reactive to muscle variations, the subject must keep its muscles active to maintain the hand closed, increasing the risk of fatigue, both physical and mental.

The introduction of PVC (Figure 4C middle) clearly changes this latter behavior and consequently mitigates the risk of fatigue. It is possible to observe several time intervals (i.e., 7.5 to 8.5 s, 9.5 to 12 s, and 14 to 17 s) where both EMG signals are low—thus the respective muscles are relaxed—but the hand reference configuration is kept closed. Unfortunately, as discussed in the section 3.2, there are intervals where PVC misinterprets pure coconctraction phenomena as motion intentions (i.e., 2 to 5 s, 12 to 14 s, and 20.5 to 22.5 s).

This motivates the introduction of *CD* and the PVC/CD method (see Figure 4C bottom), which properly detects the subject's intended actions, closing and opening just when the corresponding muscle is active, and remaining in STAY state when both EMG channels are inactive, or if the user cocontracts their muscles just to increase the stiffness. Although it is still possible to notice some very fast oscillations of the state of the FSM close to cocontractions events, when just one of the EMG is active (e.g., shortly after 14 s and before 22 s), in practice these oscillations do not affect *RC* sensibly.

## 5. Prosthesis Description

Following the above preliminary validation, the algorithms PPC and PVC/CD were implemented on the SoftHand Pro (SHP), a 19 DoF robust and functional prosthetic hand with an underactuated design that allows the actuation along the first grasp postural synergy using a single motor (Godfrey et al., 2018). Muscle activation was recorded as in the previous section. The communication with the hand was run in Simulink, while the onboard sensing was achieved using a custom electronic board (Della Santina et al., 2017).

For the cases with variable stiffness control (called PS), impedance control was implemented by adjusting online the proportional gain of the controller, called as stiffness reference and defined as in

(4) $$\begin{array}{l} RS={\left[kp\cdot Is\right]}_{ls}^{hs} \end{array}$$

being *hs* and *ls* the high and low saturations. *Is* has been previously low-pass filtered. Since the SHP has already a mechanical compliance model, which is explained in detail in Della Santina et al. (2018), the actual compliance featured by the hand is a combination of the motor compliance and the mechanical compliance of the hand (*K*_*SHP*_). As *K*_*SHP*_ increases with the relative angle between the joints of the fingers, motor stiffness is more relevant in configurations where *K*_*SHP*_ is larger, as it occurs when the hand grasp tightens around a target object.

## 6. Algorithm Feasibility in Prosthesis Users

To explore the feasibility of using our algorithm for the impedance control of a prosthetic hand, and preliminarily investigate how this could affect the use of the prosthesis during daily life (in section 7), we present a pilot test executed on a single prosthesis user. For the moment, this prevents any possibility of generalizing our results over patient variabilities that can arise due to several factors, the most important of which are the differences in their residual limbs and in their specific muscle conditions. This effort is deferred to future extensions of this study, that will include a larger and statistically significant number of amputees.

The subject involved (female, age 37, employed in the lab as administrative support, not an author of the study) has a congenital malformation at the trans-radial level of the left arm. She is a customary user of a cosmetic prosthesis, but she is also familiar with myoelectric prostheses. Although she can properly control standard myoelectric hands, with two independent antagonistic EMG signals, she is not used to include the artificial hand in the execution of ADL, or in interactions with other humans. In addition, even though natural coactivation can be found in her EMG recordings, the user has never intentionally used coactivation as a control input to command prostheses, neither for the control of impedance, nor for switching. To account for this, a training phase was performed to let the user learn the voluntary use of coactivation. All procedures (including experiments in section 7) were executed under the approval of the Ethical Committee of the University of Pisa and with informed consent from all participants.

This experiment analyses the differences in performance of four control modalities in response to external disturbances:

- **PPC-HS:** Proportional Position with High constant Stiffness;
- **PVC-HS:** Proportional Velocity with High constant Stiffness;
- **PVC-LS:** Proportional Velocity with Low constant Stiffness;
- **PVC-PS:** Proportional Velocity with Proportional Stiffness.

Following the results of section 4, all modalities with proportional velocity control (PVC) use the proposed PVC/CD method to decode user's intended actions, also in the cases where the stiffness is constant. All control systems were implemented on the same hardware presented in section 5. The implementation of the hand stiffness control, as well as the method used to estimate the grasp force, are analogous to Ajoudani et al. (2013). The first two modalities (PPC-HS and PVC-HS) correspond to the ones presented in section 5, when discussing the reconstruction of position intentions. Instead, the last three control methods (PVC-HS/LS/PS) are further explored in section 7 to focus the study on the effect of stiffness in the performance of ADL and social interaction.

### 6.1. Experimental Protocol

The user was let to familiarize with cocontraction for 5 min before starting the experiment. She was asked to perform cocontractions of different amplitudes and durations. Visual feedback of the FDS and EDC levels was being provided during the trial and the training, to assist the user in maintaining steady cocontraction levels and properly activate both muscles simultaneously.

The user was asked to grasp and hold a plastic bar (2 cm diameter and 32 cm length), in front of her. An experimenter then pulled the bar from both extremities, toward the distal side of the prosthetic hand, as to pull it away from the grasp (see Figure 3). Then, the reaction behavior was observed and reported.

In a first condition, the described action was repeated, in each of the four control modalities, with the user being instructed to not oppose to the pulling action. This condition is called “involuntary reaction,” based on the consideration (see the section 6.3 for a discussion) that it is still possible to observe some reactions of the subject-prosthesis ensemble, both at the level of user's muscles (EMG activations, due to different mechanisms, including reflexes, a study on the origin of which is out of the scope of this work), and at the level of hand motors (τ variations due to changes in the hand position and in the stiffness command).

Then, the experiment was repeated again, for the four control modalities, in a condition in which the subject was instructed to voluntary apply cocontraction before the external perturbation was applied (condition “voluntary cocontraction”). No intentional closure or aperture of the hand was executed in order to evaluate the reaction of the system with respect to cocontraction modulation only.

For each of the eight experimental conditions described, the user executed one repetition, to obtain a representative sample of subject muscle activation and not be affected by fatigue. Afterwards, to evaluate more thoroughly the possibilities offered by the proposed control method (PVC-PS), we repeated the action in the “involuntary reaction” condition for 10 trials and present its results.

### 6.2. Results

**Figure 5:**
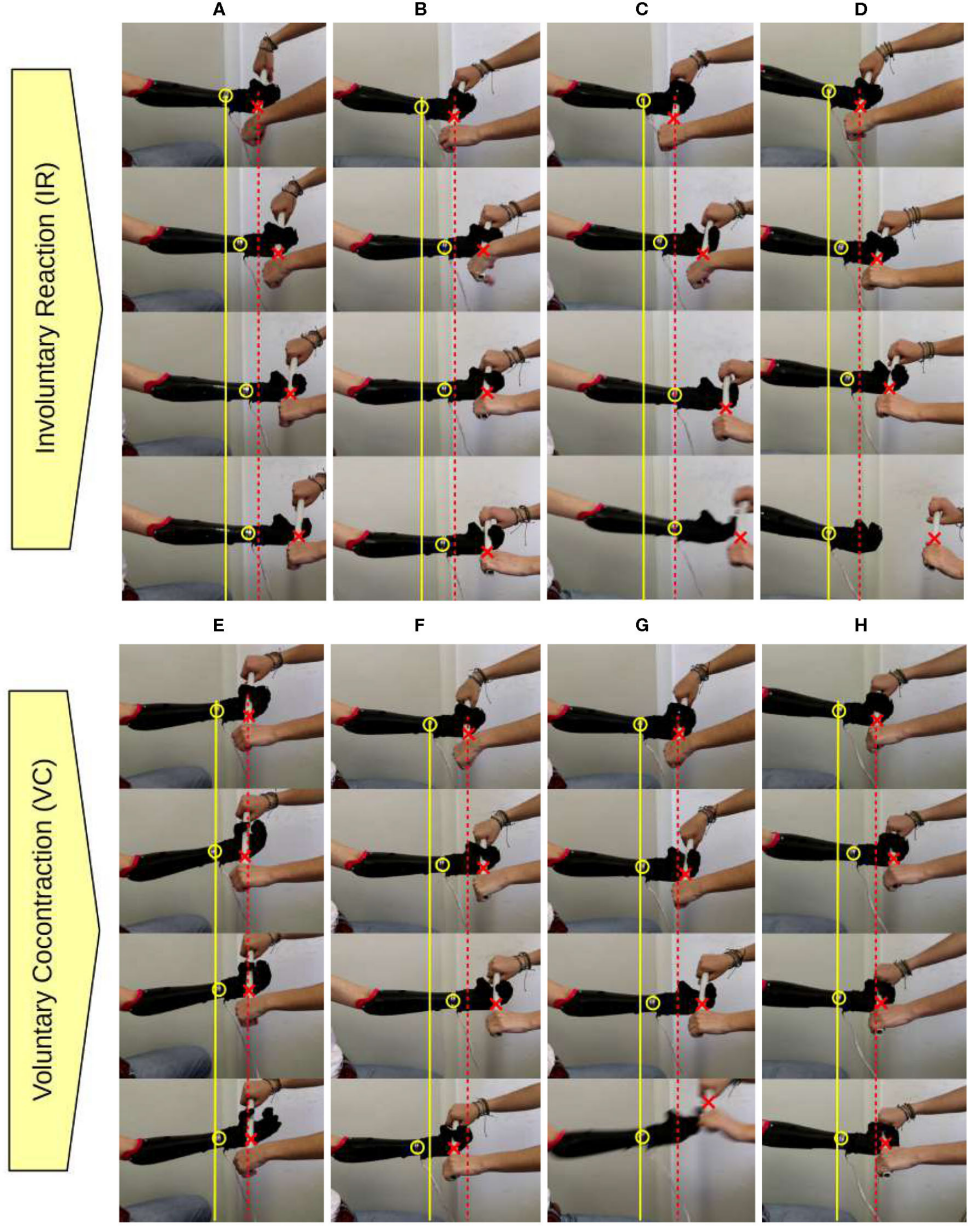
Feasibility experiment, reaction to an external perturbation. Vertical photo-sequences of the four control modalities [from left to right: **(A,E)** PPC-HS, **(B,F)** PVC-HS, **(C,G)** PVC-LS, and **(D,H)** PVC-PS] in the two conditions (IR, top and VC, bottom). A yellow circle and a red cross mark the position of the hand base and the cylinder across frames.

Figure 5 reports a photo-sequence of the eight experimental conditions. All pictures are extracted from a video recording of the experiments, obtained with the camera in a fixed position. These videos are included in the additional material together with the corresponding data synchronized. In all the frames a yellow circle and a red cross were overlaid to mark the position of the hand base and the plastic cylinder, respectively. A continuous yellow line and a dashed red line mark, across frames, the position of both in the first frame, i.e., before than the perturbation was applied.

**Figure 6:**
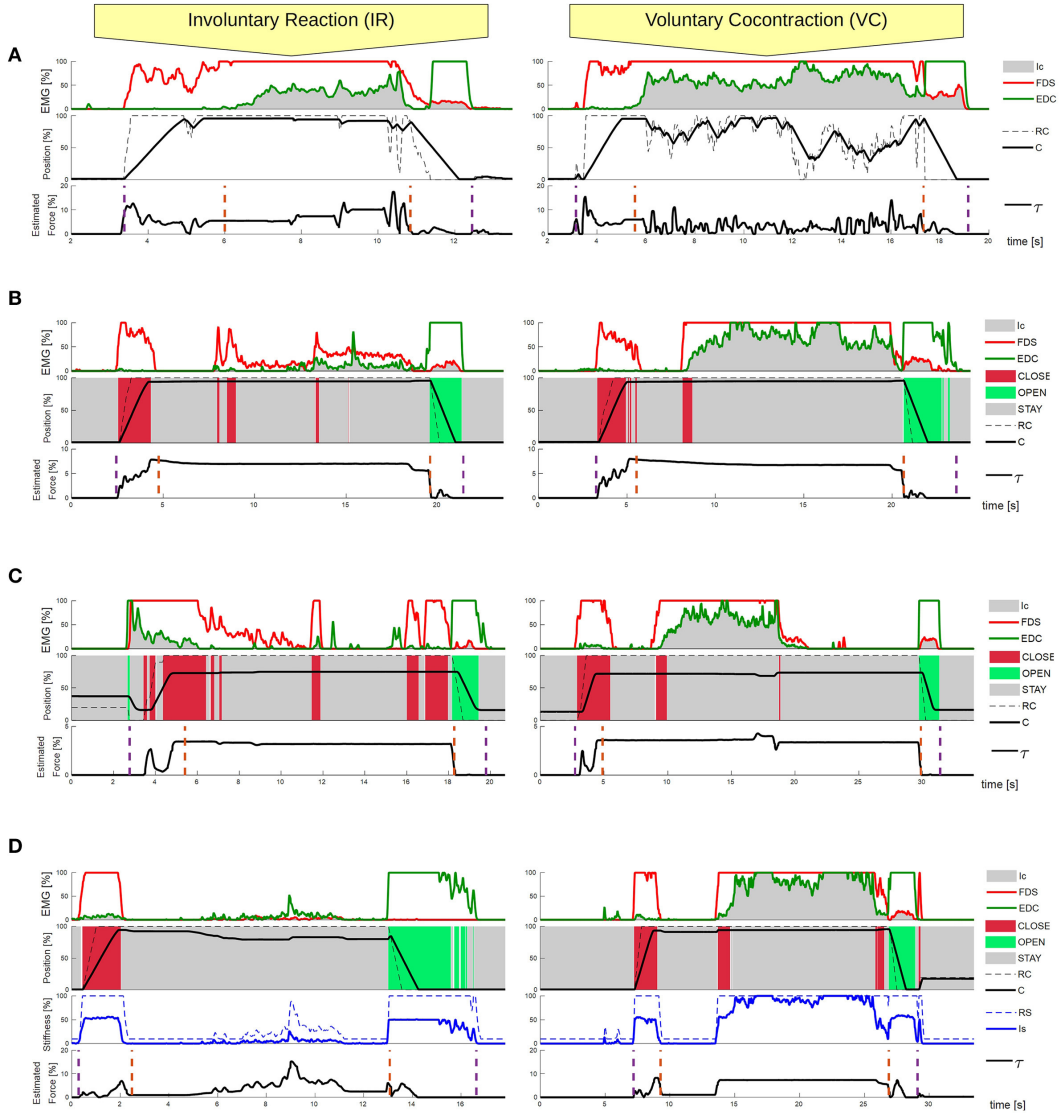
Feasibility experiment, reaction to an external perturbation, recorded data. Experimental comparison between the four different control strategies: **(A)** PPC-HS, **(B)** PVC-HS, **(C)** PVC-LS, and **(D)** PVC-PS. The performance of the system is reported in response to an external disturbance in two conditions (IR and VC). Each case provides information about the system outputs (position and estimated force) dependant to the EMG activation and stiffness reference. **(D)** Presents also the RS and Is for the stiffness of the system, as it is the only control method with variable stiffness. The purple dashed lines specify the starting and ending moments of the action, while the orange dashed lines define the grasping phase, where the object is already grasped.

Figure 6 reports the data recorded from the system. In particular, the topmost panel reports the values of the EMG signals during each experimental condition. The second panel reports the Reference Configuration *RC* as reconstructed by the different control modes, and the current position (*C*), measured by a magnetic encoder, which is part of the standard setup of the prosthetic hand. Both quantities are relative to the position of the motor that controls the synergistic closure of the prosthesis. Just in the proposed method (PVC-PS), which is the only one with variable stiffness control, the third panel displays the observed stiffness index and the final reference stiffness commanded to the system. The bottom panel for each condition reports a reconstruction of the torque (τ) that the motor exerts on the hand along its synergistic direction of motion, which is directly proportional to the force applied to the object. This is controlled (by the embedded hand controller) to be proportional to the difference between the reference command (*RC*) and the measured motor angular position (*C*), multiplied by the stiffness of the controller (*RS*), as in

(5) $$\begin{array}{l} \tau_{REF}≜RS\cdot \left(RC-C\right). \end{array}$$

Based on a model of the dynamic behavior of the controlled motor, the actual output torque can be reconstructed as

(6) $$\begin{array}{l} \tau ≜\tau_{REF}-K_{BEF}\cdot RS\cdot \left(\omega \cdot |RC-C|\right), \end{array}$$

where ω is the speed of the hand and *K*_*BEF*_ is an experimentally defined correction constant. All variables are normalized and expressed in percentage.

In each of the conditions, we mark the beginning and the end of the grasping action and the interval of grasp. The beginning and the end of the grasping action are extracted with a threshold detection on the correspondent EMG signal (start of FDS for the beginning of grasping, end of EDC for the end of the grasp). The grasping phase, where the cylinder is already grasped, is extracted from the analysis of *C* and *RC* values. In particular, when the *RC* arrives to the maximum and the difference between *C* and *RC* is constant for three samples, this suggests the success of the grasp and marks its beginning. To mark the end of the grasp, we look for when the difference of *C* and *RC* changes its sign, indicating that an intended action of opening has initiated.

From those, it is possible to extract a series of metrics characterizing the various trials, that are reported in Table 1. In particular, from the analysis of the grasping force as reconstructed in Equation (6), we can evaluate the maximum (Max. Torque) and mean (Mean Torque) during the steady-state grasping phase. For each of the two EMG signals, corresponding to the FDS and EDC muscles, we can measure the mean activation during the whole action (Total FDS/EDC) and during the grasping phase (Grasping FDS/EDC). These variables are an indirect measurement of the user's risk of fatigue, with larger values corresponding to higher risk. Moreover, we report the time used to grasp the object (Grasping time), defined as the difference in seconds between the beginning of the action and the start of the grasping phase. This measure is related to the reactivity of the hand.

**Table 1:** Results of experimental validation from Figure 6.

	**Involuntary reaction**				**Voluntary cocontraction**			
	**PPC-PS**	**PVC-HS**	**PVC-LS**	**PVC-PS**	**PPC-PS**	**PVC-HS**	**PVC-LS**	**PVC-PS**
Max torque (%)	17.50	7.70	3.45	15.28	14.15	7.87	4.27	7.38
Mean torque (%)	7.28	6.92	3.22	3.84	3.31	6.90	3.50	5.72
Total FDS (%)	75.20	24.90	39.71	10.79	87.72	68.03	44.74	67.37
Total EDS (%)	28.91	14.48	15.11	22.57	55.73	52.11	26.48	54.25
Grasping FDS (%)	97.67	20.29	31.73	1.36	99.43	79.72	42.67	72.33
Grasping EDS (%)	33.79	6.64	4.01	4.86	61.16	54.72	24.09	55.55
Grasping time (s)	2.12	1.79	2.11	1.68	1.91	1.77	1.65	1.58

**Figure 7:**
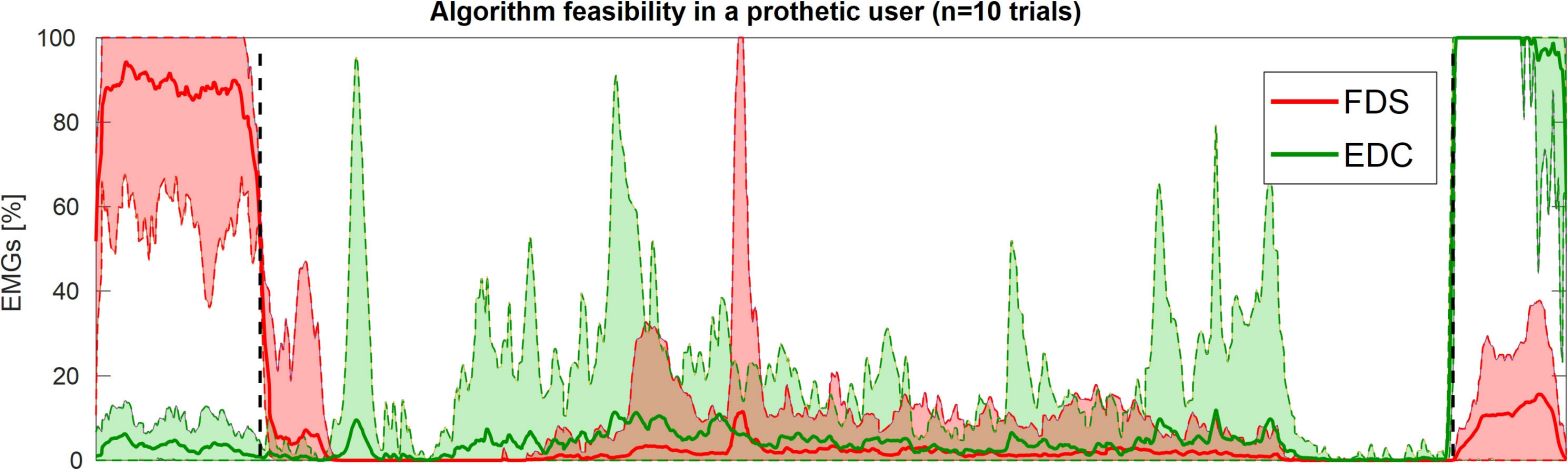
Feasibility experiment, reaction to an external perturbation, average FCS/EDC activation levels during repeated PVC-PS “involuntary reaction” experiments. Figure shows the aggregate behavior of the two EMG signals across the different trials, re-synchronized to have the three phases closing—intermediate state—opening overlap. Continuous lines represent the mean values while the dashed lines and colored area define the envelope of the maxima and minima of all signals.

Finally, Figure 7 presents the average behavior of the subject's EMG signals during ten repetitions of the condition PVC-PS (involuntary reaction). All data are synchronized to overlap the three phases of the experiment: closing—intermediate state—opening (marked with black dashed lines). The selection of the phases was done through the state-transitions of the FSM, selecting the intermediate state as the phase between the first transition from CLOSE to STAY and the subsequent first transition from STAY to OPEN.

### 6.3. Discussion

This validation confirmed that the implementation of the different control modalities make the system behave differently, as it is already appreciable in Figure 5. Performing the same action, we observe different levels of force applied to the object grasped when using alternative control modalities (see Figure 6).

The results from IR condition of the PPC-HS and PVC-HS (see Figures 6A,B), show the occurrence of involuntary cocontraction during the grasping phase and their very different effect to the two control modes. We observe how cocontraction appears more easily in PPC due to the already active FDS muscle. The hand closure is not affected in PVC-HS, as involuntary muscle coactivation is mainly interpreted in the STAY class. On the contrary, in PPC-HS, the increase of cocontraction affects also to the level of closure of the hand, which can compromise the grasp, as occurs in Figure 5A.

The effect of voluntary cocontraction in PPC-HS and PVC-HS is also presented. It is even more clear how the control of *RC* is very difficult in PPC, as the hand opens because of the excessive increase of EDC (visible both in Figure 6A (VC) and Figure 5E). Note that in PPC, high levels of cocontraction develop also into a very variable force, making the grasp uncomfortable and hard to control finely. This issue is noticeable in Figure 5E, where the user experiences problems to maintain the hand closed even before the cylinder is pulled. In the case of PVC-HS, the voluntary use of cocontraction remains robust, without affecting *RC*.

Comparing PVC-HS with PVC-LS, in both IR and VC conditions, we can appreciate the effect of different values of stiffness. We observe an almost constant value of the estimated force applied throughout the whole grasp phase, even when there are no external disturbances, and with a larger value corresponding to larger stiffness index (see Figures 6B,C). This has an effect on the experiment, while in Figures 5B,F, the hand tries to oppose constantly to the loss of the cylinder when the experimenter is pulling with a very rigid grasp, the object is easily lost in cases associated with low stiffness (Figures 5C,G) due to the softness of the grasp. Note that neither of the two behavior is, *per se*, right or wrong. Indeed, depending on the specific interaction and on the subject intentions, either of the two behaviors could be desired.

For the case with variable stiffness control (PVC-PS), we observe how the involuntary reaction to external perturbations only affects the reference stiffness and, therefore, the grasping force when required. This is visible in Figure 5D where the hand remained closed, even when the cylinder is removed. The effect of voluntary cocontraction in PVC-PS is presented in Figure 6D. Once more, we notice how the use of the PVC/CD reconstruction method makes cocontraction not affect the position command (as opposed to PPC). We can clearly see how the cocontraciton translates in an evident change of the stiffness command. As a consequence, the hand remains closed and only the stiffness increases, resisting the removal of the cylinder. In addition, a more continuous torque is applied during the grasping phase. The effect of this is visible in Figure 5H, where the experimenter is not capable of removing the cylinder. Nonetheless, it is important to note that with a variable stiffness strategy, the force decreases when the object is already grasped if no perturbation occurs.

Finally, we observe that the use of voluntary cocontraction is minimal (approximately 10 s), as the prolonged contraction of muscles is considered fatiguing by the user. However, for a PVC with variable stiffness control, it proved to be very useful to react to perturbations and adapt to different situations.

Table 1 shows that the Total FDS/EDC in IR is always smaller in PVC than in PPC. Moreover, Grasping FDS/EDC values highlight how the EMG activation can be almost zero for involuntary reaction in PVC, contrarily to PPC, and so this should sensibly reduce the risk of fatigue. Regarding the Grasping time results, we remark that similar values were recorded for PVC-HS/LS/PS. However, due to the smaller gain (*Kp*) in PVC-LS, the difference between *RC* and *C*, when the grasp is considered successful, is much larger. Moreover, although proportional position controllers (PPC) are more reactive by definition, the results show longer grasping times than in PVC-HS. This issue could be caused by the difficulties encountered to fine control the hand closure with PPC.

To sum up, with variable stiffness control, the estimated force varies along the grasp action adapting to different conditions, both external (such as disturbances), and internal (such as reflexes and voluntary changes in cocontraction). This results in a larger force and a more rigid grasp only when needed or desired. Therefore, the hand is more responsive to unexpected situations, while remaining adaptable and soft otherwise, promoting thereby human interaction and collaborative tasks. Please refer to the Supplementary Video for a detailed visualization of the system performance in each condition, synchronized with the recorded data.

Finally, in Figure 7, we observe how the user is capable of performing and using the algorithm properly in all 10 trials, while always having a certain involuntary muscle reaction during the intermediate state (probably at the beginning of the external perturbation, which was different in each condition). Indeed, the user got an absolute intermediate activation—volume expressed in mean (SD)—of FDS = 2587.4 (3090.7) and EDC = 5719.8 (4862.5), much lower than the voluntary activation for the intended actions of close and open, but still appreciable for the system.

These repetitions reinforce the feasibility of the algorithm and the need of a myoelectric algorithm that understands better external situations and user intentions, and adapt the system accordingly, as the one we proposed. Although the present work does not provide evidence on the extendibility of our results to other prosthesis users, the positive outcomes suggest further investigations, especially in subjects with similar muscle conditions. These would be useful toward studying generalization to different patients, effects of fatigue, attentional requirements, and embodiment.

## 7. ADL and Social Interaction

The previous protocol helped the user in familiarizing with the capabilities of the various control modalities implemented in the SHP. This section compares different stiffness control modalities to explore the potential of variable stiffness in some Activities of Daily Living, that include self-interaction and social interaction. The compared control modalities are: high constant stiffness, low constant stiffness, and variable stiffness (proportional to muscle coativation). Since the previous section confirmed the poor performance of PPC, both in terms of risk of fatigue and control finesse, only PVC is used to control joint position in all conditions. Accordingly, the different behaviors experienced during the experiment are mainly related to different stiffness behaviors.

The ultimate purpose of this investigation is to evaluate the needs of prosthesis users, in terms of hand stiffness modulation, when performing different types of tasks. Therefore, despite being aware of the limitations of performing this pilot study with just one user, we consider the opinion and experience of a prosthesis user very valuable. Moreover, we analyse the preferences and opinion of able-bodied subjects that interact with the user as secondary subjects. The authors believe that it is also important to know what people expect or want when interacting with robotic hands to improve and normalize their acceptance in society.

To account for the variability in human interaction preferences, the experimental protocol is repeated with 12 secondary subjects. These secondary subjects are limb-intact people (ages between 24 and 32, 7 males and 5 females) with normal motor and cognitive functions, able to understand the experimental procedure. The same prosthesis user presented in section 6 participated in this study. All subjects (1 limb-loss + 12 limb-intact) gave informed consent.

Our hypothesis is that a stiffer control may result in a very rigid grasp, which can cause difficulties in bimanual or collaborative tasks. However, the same modality can improve user's reliability when moving heavy objects, where a very strong grasp is needed in order not to lose the item. Likewise, the authors hypothesize that a softer control would be perceived as safer and more comfortable, and increase the overall quality of the social and self-interaction.

### 7.1. Experimental Protocol

**Figure 8:**
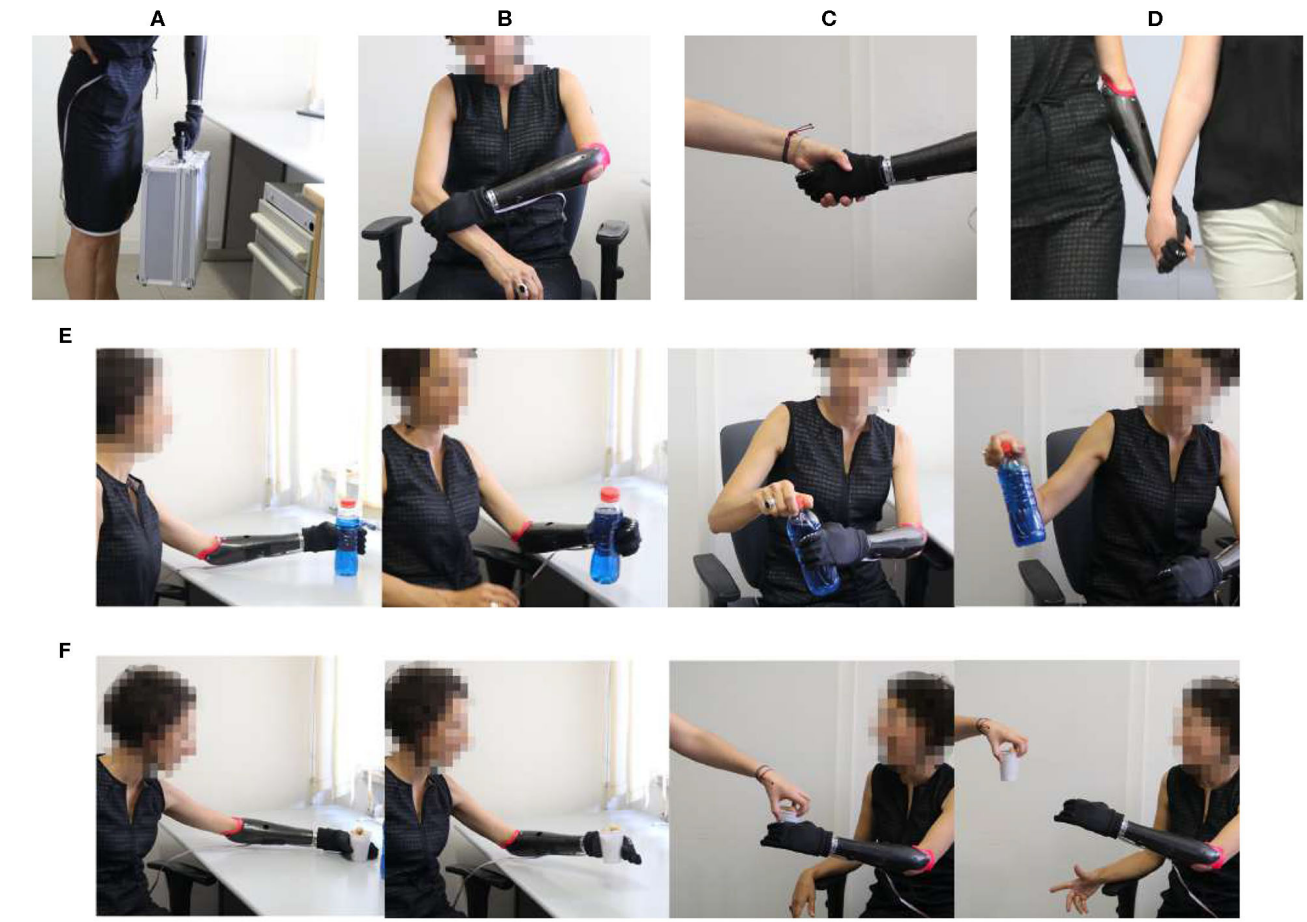
Experimental tasks. Lifting a heavy object **(A)**, self-interacting **(B)**, Hand-shaking **(C)**, walking holding hands with another subject **(D)**. In **(E)**, the user performs a bimanual manipulation of a bottle of water and in **(F)** the user is passing a fragile object to another person.

The prosthesis user is asked to execute a sequence of tasks, designed to require different amount and modulation of grasping force involved. The experimental tasks are divided in two groups. The first group of tasks is performed by the prosthesis user alone (see Figures 8A,B,E):

**T1**. Lifting a heavy object (full tool case) from the floor to the table;
**T2**. Self-interaction (e.g., squeeze the sound arm with the prosthesis, perform hand-shaking with herself or trying to open the robotic hand when closed without active opening thought EMG sensors.)
**T3**. Bimanual manipulation: grasping several objects characterized by different properties (heavy, light, and fragile) with the prosthetic hand, pass them to the intact hand, and then release them.

The second group of tasks foresee physical interaction with the secondary subjects. These tasks allow us to study the perceived effects of the various controllers in physical social interactions between humans, mediated by a robotic prosthesis. They are:

**T4**. Hand-shaking (see Figure 8C);
**T5**. Objects passing: the same group of objects from T3 were used to evaluate system's collaborative performance (see Figure 8F);
**T6**. Walking hand in hand with another participant (see Figure 8D).

The prosthesis user was required to speak during the execution of tasks involving bimanual actions, T3 and T5 (Figures 8E,F). In this way, we forced the user not to pay excessive attention to the moment where the object goes from the prosthetic to the intact hand, imitating the natural way humans interact. An example of the effect of this was observed in task T5, where the prosthesis user did not actively release the object, but held it with a looser grasp and let the secondary subject take the object without hindrance. The user did not receive explicit instructions about the use of cocontraction during the experiment. As such, arising cocontraction phenomena were mostly involuntary, reflecting the natural reflex of user's muscles in different situations (e.g., they tended to occur when moving heavy objects, while they did not happen when interacting with light objects or with people). The nature and size of the objects involved in T3, T5, and T1 are the following:

- Plastic cups (200 ml) of 2.4 g,
- Full plastic bottle (0.5 L) of 0.5 kg,
- Metallic tool case (395 × 300 × 135 mm) of 4 kg.

Two of the three plastic cups were filled with wooden balls (d = 1.8 cm of 0.8 g. per ball) to slightly increase their weight. One of the cups was completely full of balls, while the other one was half-full.

For each secondary subject the experimental protocol was repeated with the three different control modalities. Subjects (prosthesis user and secondary subjects) were naive about the modality implemented on the system. The presentation order of the control strategies was randomized by blocks in order not to favor any possible learning. Moreover, this randomization increases the difficulty for the prosthesis user to recognize the control properties over iterations. Inside of each block, the order of the objects presented in T3 and T5 was also randomized for each subject to avoid the learning of the force required to move the objects.

As underlined in Biddiss and Chau (2007), standard outcome measures are very important to better understand the impact of proposed solutions in prosthesis use and abandonment. To evaluate control systems performance and the interaction perception, we asked the subjects in charge of system's evaluation to answer two different tests right after each experimental case.

Only the prosthesis user is required to compile the System Usability scale (SUS) (Brooke et al., 1996). Similar to other client-rating outcome measures (e.g., TAPES-R, DASH—Wright, 2013), SUS presents evidences about control systems usability perceived by the user. The questions are reported together with results in Table 2.

**Table 2:** Results of the System Usability scale from a prosthesis user.

**Scale (1 Strongly disagree - 5 Strongly agree): mean±SD**			
**Questions**	**HS**	**LS**	**PS**
Q1*. I think that I would like to use this system frequently	2.67 ± 0.94	1.92 ± 0.76	4.25 ±0.92
Q2*. I found the system unnecessarily complex	2.75 ± 0.43	3.67 ± 0.47	2.25 ±0.72
Q3*. I thought the system was easy to use	3.58 ± 0.64	2.33 ± 0.85	4.08 ± 0.76
Q4. I think that I would need the support of a technical person to be able to use this system	2.92 ±0.28	2.83 ± 0.37	2.75 ± 0.43
Q5. I found the various functions in this system were well integrated	3.00 ± 0	3.00 ± 0	3.08 ± 0.28
Q6. I thought there was too much inconsistency in this system	3.00 ± 0	3.00 ± 0	3.00 ± 0
Q7*. I would imagine that most people would learn to use this system very quickly	3.75 ± 0.43	3.00 ± 0.71	3.83 ± 0.37
Q8. I found the system very cumbersome to use	3.08 ± 0.28	3.17 ± 0.37	3.25 ± 0.60
Q9*. I felt very confident using the system	2.42 ± 0.76	1.75 ± 0.43	3.67 ± 0.75
Q10. I needed to learn a lot of things before I could get going with this system	2.75 ± 0.43	2.83 ± 0.37	2.58 ± 0.49
SUS score* (Two-way ANOVA)	52.29 ± 5.05	41.25 ± 5.89	62.71 ± 7.72

*Color green indicates the modality with better results for significant questions*.

Inspired by a similar study on human-robot interaction (Vigni et al., 2019), four questions were asked to the able-bodied subjects in order to rate the interaction quality, human-likeness, safety, and comfort on a likert-scale. As no feedback was given to the user while interacting with the secondary subjects, we did not ask the prosthesis user to respond the same questions as secondary subjects, as results would not be comparable. Rather than quantitative data, we report qualitative data from free comments that still inform about user's perception of the interaction.

### 7.2. Results

Due to the dataset being discrete and not normal (Lilliefors test), we applied Friedman ANOVA test for pairwise non-parametric data. In the case of secondary subjects, the opinion between control modalities was dependent (pairwise), while for the SUS test was also considered dependent, even if all answers came from the same prosthesis user, as we found differences at the SUS data distribution over time. ANOVA test informs whether there is an overall difference between groups (control modalities), but it does not tell which specific group differed from others—*post-hoc* tests do. After Friedman, *post-hoc* adjustments were applied, particularly Tukey's honest significant difference (HSD) criterion, only to the questions that showed an overall statistically significant difference from Friedman ANOVA.

We feature Tables 2, 3 that present information about the results (mean±SD). Table 2 reports the opinion of the prosthesis user, while Table 3 presents the average qualification from the 12 able-bodied subjects. Color green indicates the modality with better results, highlighting only questions with statistical significance resulting from Friedman ANOVA.

**Table 3:** Results of the questionnaire about Human-Prosthesis interaction from 12 secondary subjects.

**Questions**	**Scale (1–5): mean±SD**	**HS**	**LS**	**PS**
Q1	Very poor to very good quality	3.75 ± 0.83	4.25 ± 0.60	3.92 ± 0.86
Q2*	Very robot-like to very human-like	2.92 ± 1.04	3.92 ± 0.64	3.25 ± 0.83
Q3	Very unsafe to very safe	3.75 ± 0.72	4.25 ± 0.60	4.00 ± 0.71
Q4*	Very uncomfortable to very comfortable	3.17 ± 0.80	4.08 ± 0.64	3.25 ± 0.92

*Color green indicates the modality with better results for significant questions*.

**Figure 9:**
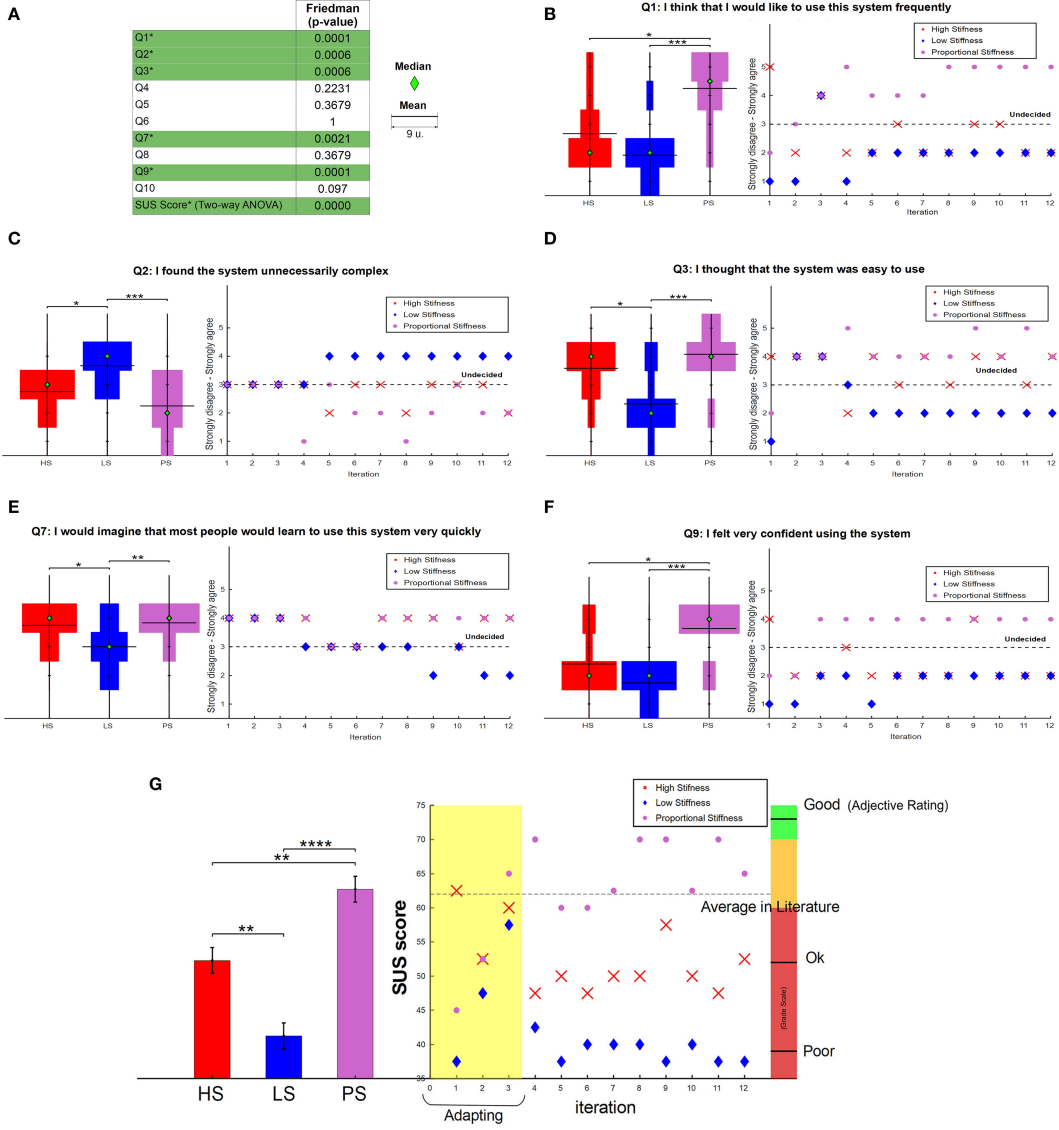
Prosthesis user SUS test. **(A)** Reports *p*-values and a single legend, where the median is represented by a green diamond and the mean by an horizontal black line (the size of the line of the mean refers to 9 units). **(B–F)** Report data distribution (left) and answers evolution (right) for statistically significant questions. On the left, a discrete Violin graph presents the distribution of the answers for the three control conditions. Statistical significance is expressed by asterisks, where *p ≤ 0.05, **p ≤ 0.01, ***p ≤ 0.001, and ****p ≤ 0.0001. On the right, a scatter plot of the data over iterations. **(G)** Reports the estimated means and standard errors (left) and the evolution trend (right) of the SUS score.

The Friedman *p*-values obtained for all SUS questions are reported in Figure 9A. Figure 9 presents data distribution (left of each subfigure) for the SUS questions that resulted significant, i.e., Q1, Q2, Q3, Q7, and Q9. Significances across multiple tests are expressed through asterisks between control modalities (coming from Tukey's honest significant difference). There, Q1 and Q9 present significance between the same control modalities. Likewise, the same thing occurs in Q2, Q3, and Q7. In addition, we present the evolution of the SUS answers over iterations on the right side (see from Figures 9B–F).

Please, note that the last raw of Figure 9A shows statistical significance also in the overall SUS score, as a result of a Two-way ANOVA test instead of Friedman, due to the continuous nature of SUS score values. Figure 9G presents the estimated means and standard error from SUS score among control systems and its time-evolution. It proves the preference of variable stiffness control (PS) over the other two options with a value close to the average in literature to be accepted. Free comments of the user were also collected, and a summary is reported in the discussion, in section 7.3.

**Figure 10:**
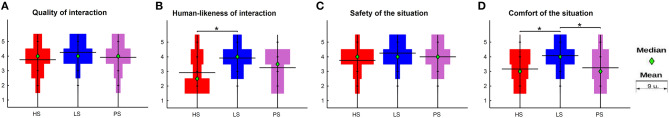
Secondary subjects questionnaire. Discrete violin plot presents the distribution of answers obtained to different questions of the human-prosthesis interaction. Friedman ANOVA test *p*-values: **(A)** 0.2956, **(B)** 0.0366, **(C)** 0.1353, and **(D)** 0.0117. Statistical significance between control modalities is expressed by asterisks, where *p ≤ 0.05. A single legend is presented on the right side, where the median is represented by a green diamond and the mean by an horizontal black line (the size of the line of the mean refers to 9 units).

The same statistical analysis of Friedman + Tukey's was done to the results from secondary subjects, where Figure 10 shows the data distribution of all four questions asked. The *p*-values from Friedman test are detailed in the caption, while significance between control modalities is presented through asterisk after the *post-hoc* test, directly on the plots. Questions Q2 and Q4, which refer to “human-likeness” and “comfort” presented significance with favorable results for low stiffness.

### 7.3. Discussion

The fact that we obtained statistical significance, both in primary and secondary questions, presents evidences in favor of the hypothesis that the different strategies implemented influence subjects' perception. Moreover, we can also infer the difficulties on task execution from the differences in performance.

Looking at the evolution of the SUS answers, it is possible to appreciate a trend which could suggest the existence of an adaptation process. This could be due to the task learning, or to the fact that the user, over iterations, learned to recognize the controllers and thus adapt faster to them. We observe that the user started preferring both high constant stiffness (HS) and proportional stiffness (PS) due to their reactivity, which feels more natural and personal as the hand responds faster to muscles commands. However, this changed over the course of the experiments when the possibility of causing discomfort to people, due to an excessive squeezing with HS, arose. The user also underlined the lack of confidence and difficulties to command the hand when using the low constant stiffness controller (LS), because of the opposite reason, i.e., lack of reactivity (see Figures 9C–E). Finally, PS was perceived as the easiest system to use and learn during the whole test, in accordance with the fact that it is designed with different stiffness requirements and adapts to situations. Figures 9B,F prove the user's preferences in use and confidence with a high significance against the other two control modalities.

Without explicit knowledge about the controller used, the user was able to understand the differences in performance and the diverse sensations elicited through the experiment. This perception fits with our hypothesis, validating the proper implementation of variable stiffness control. The user reported the following comments:

**HS:** “*The grasp is very rigid.” “The grasping force could be dangerous when interacting.” “I am scared of hurting people, I do not like this control. I feel like it is my fault and I need to learn how to measure the force executed.” “I do not feel very confident when interacting with people.”*;

**LS:** “*It has a smooth grasp. When holding something, the fingers are not rigid.” “The motion is very slow. The hand does not close enough.” “The grasp feels inconsistent, unreliable and uncomfortable.” “I need to use a lot of muscle force to activate it, but the result is always imprecise. I do not like it and it is fatiguing.”*;

**PS:** “*The grasp was firm but adaptable/flexible while keeping the object grasped.” “I prefer this control, even if I grasp with a lot of force, I feel safe and secure (also in human interaction) as the hand is adaptable when I am relaxed.” “I feel confident, even if the hand is closed, if someone or something forces it to open, it opens a bit.” “It allows human-like interaction*.”

Finally, variable stiffness SUS score obtained a higher acceptability and usability over both constant stiffness control modalities. Moreover, Figure 9G presents the evolution of the SUS score over iterations, from which we can observe how, from the 4th iteration on, user's preferences start to be more or less constant. While variable stiffness is close or over the average in literature, low and high stiffness got lower scores of “poor” and “ok,” respectively.

As observed in the seminal work by Hogan (1983), and proved by the diffusion of modern impedance controlled robots, it is well-known that one of the situations in which impedance control improves substantially the quality of interaction is force control with positioning errors. The condition in which a prosthesis user pilots their device is very similar, because of the poor proprioceptive feedback (which is usually solely visual). Indeed, also in our experiments, secondary subjects seem to perceive impedance control better than high constant stiffness control. This is hinted to in Table 3 where in all of the questions the proportional control (PS) rates between HS and LS, although the difference is not significant. This suggest that variable stiffness makes the prostheses capable of adapting better to the hands and movement of the other subjects, an aspect that was already observed by Vigni et al. (2019) in a human-robot handshaking scenario.

About the opinion and perception of the secondary subjects, we found statistical significance looking at “human-likeness” and “comfort” aspects (see Figure 10). There, low constant stiffness is significantly better than high constant stiffness. Moreover, it is worth mentioning that the force applied is affected both by the reference configuration and the stiffness, and it could be different in each case. These two factors play an important role on how subjects perceive the grasp. However, while the grip stiffness is different between HS and PS, the control of the reference configuration resulted complex to the user because of the lack of sensory feedback. Proprioception plays a critical role in enabling humans to precisely control their movements. In prosthetics, the lack of proprioception makes users rely heavily on visual feedback (Blank et al., 2008). This can be inconvenient or even impossible for some tasks, as when socially interacting. Therefore, the authors consider that the difficulties in controlling the reference configuration could have been affecting a lot on the sensation of discomfort perceived by the secondary subjects, in the cases where they considered the closure of the hand excessive; even if later the hand became adaptable when PS was implemented. This idea is notable in the “comfort” aspect, where both high constant stiffness and proportional stiffness present significant difference from low stiffness, as visible in Figure 10D.

Analysing the video footage of the 36 experiments, it is possible to observe that, when holding the tool case with a low stiffness control, the user encounters difficulty because of the loose grasp. On the contrary, when interacting with another subject using the high stiffness control, the user presents more problems to maintain a conversation due to the cognitive effort used to control the hand so not to exert excessive forces on the other subject. Both these issues were absent in the case of the variable stiffness control, where the user is able to move the tool case with ease and interact while seamlessly having a conversation, suggesting a good control and a low cognitive load. Interestingly, this effect is also visible in bimanual manipulation, where the prosthetic hand adapts better to external conditions when using variable stiffness, allowing the user to move the objects faster while having a more fluent conversation.

**Figure 11:**
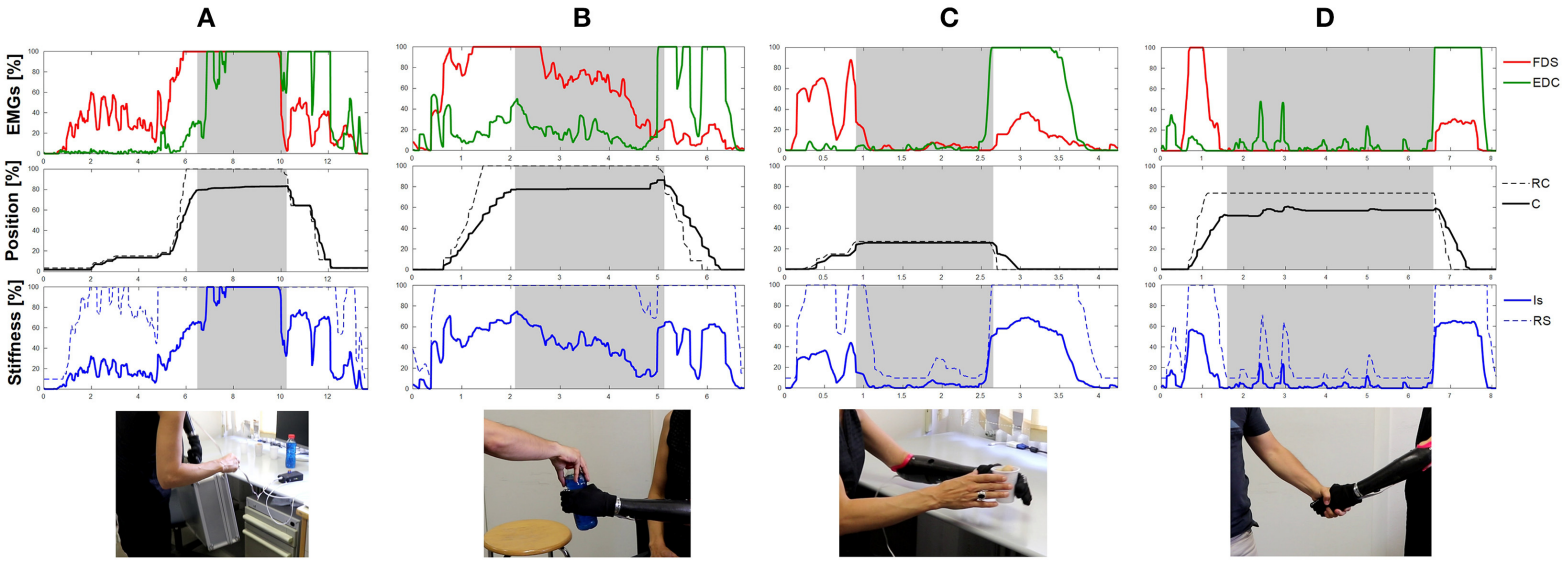
Data recorded during the experiment. Natural and involuntary reaction occurs in user's muscles depending on external conditions. Here, **(A)** presents an example of the user's muscle when moving the tool case, **(B)** shows user's data when interacting with a bottle full of water, **(C)** when interacting with a light and fragile plastic glass, and **(D)** when hand-shaking with a secondary subject.

Furthermore, Figure 11 presents examples of the data recorded during these experiments. There, we can observe how, as hypothesize and discussed in previous sections, user's muscles adapt to the external conditions intuitively as muscles of healthy humans do. Indeed, Figure 11A (Task T1) shows a clear and very strong cocontraction during the holding and moving phase of the tool case, but only variable stiffness control benefits from this modification and modify the stiffness of the prosthesis in accordance. Note that with the same muscle conditions, PPC or a bad FSM for PVC would not be capable of understanding the requirements of the situation, hindering the performance of the user. Figures 11B,C (T5 and T3, respectively) show how even with lower weights (plastic bottle), the effect of muscles adapting to external loads is visible in bimanual interaction. Finally, Figure 11D (Task T4) highlights the absence of muscle contraction when interacting with humans or soft materials, which results in a low stiffness during the grasping phase, with more adaptable fingers. Please refer to the Supplementary Video for a detailed visualization of the executed tasks, synchronized with the recorded data.

The number of healthy subjects involved in the interaction experiments and the number of repetitions performed allow us to confidently affirm that, for this user, the method is certainly feasible and indeed, very appreciated. Nevertheless, given the involvement of only one prosthesis user in the study, it is not possible to generalize our results.

## 8. Conclusions

In order to achieve different desired behaviors in upper limb prosthetics for Activities of Daily Living and for social interaction, an alternative solution to the classical sEMG based control is explored. The proposed method includes stiffness modulation of the hand, proportional to muscle coactivation, with a proportional velocity control of hand position that includes a finite state machine. The feasibility of the algorithm is preliminarily validated with a prosthesis user, comparing its performance with other conventional control modalities implemented in the SHP. Eventually, this concept could be implemented in other rigid prosthetic hands, where differences between modalities could be even more visible/useful, as their only compliance can be given by the motor impedance. Moreover, an important limitation during the ADL experiments was that no form of feedback was given to the prosthesis user during tasks execution. Even though impedance control is a method to manage human interaction without relying excessively on sensory feedback (Hogan, 1983), results suggest future studies about the effect of haptic feedback in social interaction.

Encouraging results suggest the feasibility of the proposed approach and a good adaptive capacity, which will be investigated more in depth in the future, with different applications and more participants. Although there is room for improvement, the authors believe in the potential of variable stiffness control implementation for prosthetic devices. Finally, as we obtain an extra available signal (proportional to cocontraction), it could be potentially used to explore the stiffness control of additional joints, e.g., a wrist, and thereby, increase the dexterity of a prosthesis.

## Data Availability Statement

The datasets generated for this study are available on request to the corresponding author.

## Ethics Statement

The studies involving human participants were reviewed and approved by Institutional Review Board of University of Pisa. The patients/participants provided their written informed consent to participate in this study. Written informed consent was obtained from the individual(s) for the publication of any potentially identifiable images or data included in this article.

## Author Contributions

PC-M, MC, and GG designed the proposed control method and conceived the study. All of the author contributed to the experimental design. PC-M and CP performed the experiments. PC-M executed literature research and performed statistical analysis of the database. PC-M and GG wrote all sections of the manuscript. AB contributed expertise and advice. All authors contributed to manuscript revision, read, and approved the submitted version.

## Conflict of Interest

The impaired subject, who is not an author of this paper, is employed in the lab's institution as administrative support with the task of managing project dissemination and studying the impact of prostheses on subjects and the Society. She is not involved in the development of the prosthesis functions and control. The authors declare that the research was conducted in the absence of any commercial or financial relationships that could be construed as a potential conflict of interest.
